# Atrial and Sinoatrial Node Development in the Zebrafish Heart

**DOI:** 10.3390/jcdd8020015

**Published:** 2021-02-09

**Authors:** Kendall E. Martin, Joshua S. Waxman

**Affiliations:** 1 Molecular Genetics, Biochemistry, and Microbiology Graduate Program, University of Cincinnati College of Medicine, Cincinnati, OH 45267, USA; marti4ke@mail.uc.edu; 2 Molecular Cardiovascular Biology Division and Heart Institute, Cincinnati Children’s Hospital Medical Center, Cincinnati, OH 45229, USA; 3 Department of Pediatrics, University of Cincinnati College of Medicine, Cincinnati, OH 45267, USA

**Keywords:** zebrafish, heart development, atrium, sinoatrial node, congenital heart defects

## Abstract

Proper development and function of the vertebrate heart is vital for embryonic and postnatal life. Many congenital heart defects in humans are associated with disruption of genes that direct the formation or maintenance of atrial and pacemaker cardiomyocytes at the venous pole of the heart. Zebrafish are an outstanding model for studying vertebrate cardiogenesis, due to the conservation of molecular mechanisms underlying early heart development, external development, and ease of genetic manipulation. Here, we discuss early developmental mechanisms that instruct appropriate formation of the venous pole in zebrafish embryos. We primarily focus on signals that determine atrial chamber size and the specialized pacemaker cells of the sinoatrial node through directing proper specification and differentiation, as well as contemporary insights into the plasticity and maintenance of cardiomyocyte identity in embryonic zebrafish hearts. Finally, we integrate how these insights into zebrafish cardiogenesis can serve as models for human atrial defects and arrhythmias.

## 1. Introduction

All vertebrate hearts comprise the same fundamental structural units: the atrial and ventricular chambers, which are specialized to reflect their functions receiving and expelling blood, respectively. The coordinated assembly of these chambers into a functional organ is an intricate process that is critical for normal embryonic development and throughout life of all vertebrates. In humans, mutations in genes that disrupt chamber formation and maintenance are associated with congenital heart defects (CHDs), which are the most common type of birth defect [1,2,3,4,5,6,7,8,9,10]. Many of these signals affect proper development of the atrial chamber and its ability to function, with atrial septal defects (ASDs) making up as much as 20% of CHDs found in children and adults [5,6,7,8,9]. Moreover, mutations affecting development of the sinoatrial node (SAN) can result in cardiac pacing defects, such as sick sinus syndrome, arrhythmias, and atrial fibrillation [11,12,13,14,15].

While proper blood circulation is necessary in all vertebrate organisms, the number of atrial and ventricular chambers within vertebrate hearts can vary. Mammals have a four-chambered heart with two atria and two ventricles, which are necessary for circulation to the lungs and the rest of the body. However, teleosts, such as the common model zebrafish, have a relatively simple two-chambered heart consisting of only a single atrium and ventricle. Despite these differences, the underlying molecular signals and morphogenetic processes, particularly those that govern the specification and maintenance of cardiac chambers during heart development, are conserved across species. Moreover, due to their external development, the ease of genetic manipulation, and their ability to survive the first week post fertilization without a functional cardiovascular system, zebrafish have become an excellent tool for understanding the early processes underlying vertebrate cardiogenesis [16,17,18,19,20].

Proper heart function relies on the coordinated contraction of the atrial and ventricular chambers. Importantly, zebrafish cardiomyocytes share key electrophysiological properties with mammalian cardiomyocytes, allowing them to also serve as a model for human conduction defects [16,21]. Cardiac contraction is initiated by a population of specialized cardiomyocytes in the SAN, which is located at the base of the right atrial chamber in mammals and the venous pole of the single atrial chamber in zebrafish. The electrical signal then propagates across the atrium, where it pauses at the atrioventricular canal (AVC) before moving rapidly across the ventricle [22]. The cardiomyocytes within the ventricular and atrial chambers and the SAN are functionally distinct with characteristics conferred by the expression of specific gene programs, contractile proteins, and ion channels [23,24,25,26,27]. The thin-walled atrium possesses cardiomyocytes that have a squamous morphology, disorganized sarcomeres, and a triangular action potential [22,23,24,25,28,29]. Cardiomyocytes of the thick-walled, highly trabeculated ventricle are more cuboidal, with organized sarcomeres, and a flat action potential plateau [22,24,25]. SAN cardiomyocytes have a slower rate of depolarization and are able to generate spontaneous action potentials [22,26,30,31]. In this review, we highlight mechanisms governing the development of the atrium and the SAN within the venous pole of the zebrafish heart and explore how information from the zebrafish can be used as a model to study human CHDs and arrhythmias.

## 2. Mechanisms of Atrial Chamber Development and Chamber Identity Maintenance in the Zebrafish Heart

### 2.1. Cardiac Progenitor Location and Morphology of the Developing Zebrafish Heart

**Figure 1:**
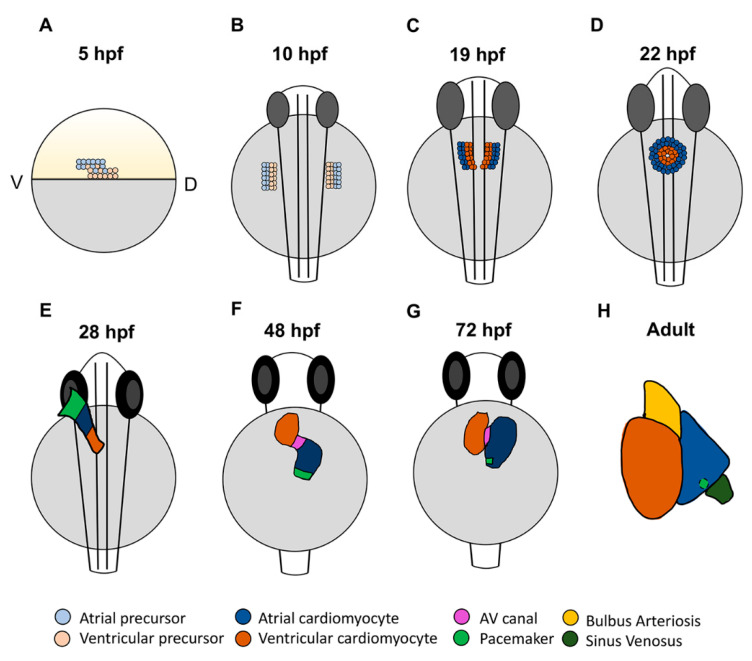
Stages of zebrafish heart development. (**A**) At 5 h post-fertilization (hpf), cardiac progenitors are located in the lateral marginal zone, with atrial progenitors located more ventrally than ventricular progenitors. (**B**) Following gastrulation at the tailbud stage (10 hpf), cardiac progenitors migrate to the anterior lateral plate mesoderm (ALPM). (**C**) In the ALPM, progenitors begin to differentiate and express chamber-specific genes. (**D**) Cells then migrate to the midline and fuse, forming the cardiac disc where atrial cardiomyocytes surround ventricular cardiomyocytes. (**E**) The disc elongates to form the linear heart tube, which begins beating by 24 hpf. At 28 hpf, the dominant pacemaker covers a large area at the venous pole. (**F**) By 48 hpf, the heart has finished looping and the two chambers have formed. Here, the pacemaker is a ring at the venous pole. (**G**) By 72 hpf, the dominant pacemaker is restricted to a small population of cells in the inner curvature at the venous pole of the atrium. (**H**) In the adult heart, the bulbus arteriosus and sinus venosus, which serve as the outflow and inflow tracts, respectively, have matured. The dominant pacemaker is located at the sinus venosus–atrial junction.

Like all vertebrates, the zebrafish heart is the first organ to form and function during embryogenesis. The initial stages of zebrafish heart development involve the specification, migration, and differentiation of cardiomyocyte progenitor cells as they are integrated into the forming heart tube [18,19,32,33,34]. Cell lineage tracing studies with caged-fluorescein have produced fate maps of the early zebrafish cardiac progenitors prior to gastrulation, which show that precursors for both atrial and ventricular cardiomyocytes first reside in bilateral populations in the lateral marginal zone by 5 h post-fertilization (hpf) [32,35]. Within the late blastula embryos, atrial cardiomyocyte progenitors are located more ventrally and slightly farther from the margin, the region of the embryo where cells will involute during gastrulation. Ventricular cardiomyocyte precursors are located more dorsally and closer to the margin. While there is a small region of overlap, these progenitor populations appear to be largely distinct; labeled cells predominantly give rise to only atrial or ventricular cardiomyocytes [32,33,35] (Figure 1A). During gastrulation, cardiomyocyte progenitor cells within the lateral mesoderm involute and migrate anteriorly to form bilateral progenitor fields in the anterior lateral plate mesoderm (ALPM) (Figure 1B). As indicated from the earlier fate maps, lineage tracing of the ALPM at later stages suggest that atrial and ventricular cardiomyocyte precursor populations retain predominantly adjacent positions: ventricular progenitors reside more medially and atrial progenitors lay more laterally [35]. Once in the ALPM, the cardiac progenitors begin to differentiate, as indicated by the expression of chamber-specific myosin genes. Expression of *ventricular myosin heavy chain* (*vmhc*), also referred to as *myosin heavy chain 7* (*myh7*), can be detected in the medial ventricular progenitors as early as the 13 somite stage [36]. Expression of *atrial myosin heavy chain* (*amhc*), also named *myosin heavy chain 6* (*myh6*), begins slightly later at 19 somites [33] (Figure 1C). While different myosins are some of the gene expression differences that define atrial and ventricular cardiomyocytes, one study has indicated that left–right asymmetry, reminiscent of the left and right atrial chambers in mammals, can also be seen as early as the 19 somite stage [37]. It was shown that *pitx2c* and *meis2b* are upregulated on the left and downregulated on the right in the zebrafish atrium, consistent with the left atrial expression pattern of *Pitx2* in mammals [37,38]. As the cardiac progenitor cells are differentiating, they migrate to the midline, begin to fuse more posteriorly (Figure 1C), and then form the cardiac disc in which differentiating atrial cardiomyocytes lie peripherally to ventricular cardiomyocytes (Figure 1D).

Importantly, vertebrate hearts are formed from the progressive differentiation of progenitor populations that grow the heart at the poles. Thus, the zebrafish cardiac disc then elongates over the yolk to form the linear heart tube (Figure 1E). The earlier-differentiating cells that comprise the initial heart tube are termed the first heart field (FHF) [39,40]. Populations of later differentiating cells, referred to as the second heart field (SHF), then contribute to both the venous and arterial poles as the heart tube elongates and grows [41,42,43]. For comparison, in mammals, the SHF contributes to the right ventricle, while the left ventricle is primarily derived from the FHF. Although both heart fields contribute to the left and right atrial chambers, it has been suggested that as many as two thirds of atrial cardiomyocytes are derived from the SHF [44]. In zebrafish, the SHF primarily contributes to the outflow tract and half of the ventricle, with a small number of posterior SHF cells populating the venous pole of the atrium [18,34,42,45]. As the heart tube elongates, it migrates left and begins looping, where by 48 hpf the physically distinct chambers have formed: the venous left-sided atrium and arterial right-sided ventricle are easily distinguishable and separated by the AVC [18,34] (Figure 1F). From this point, the heart will continue to mature and develop the additional structures that are characteristic of each chamber [19,46] (Figure 1G,H). Trabeculation in the ventricle and valve leaflet development are evident by three days post-fertilization (dpf). The mature ventricle comprises a cortical cardiomyocyte and deeper layer that make up the trabeculae, which form through the delamination of cardiomyocytes from the compact layer beginning at 3 dpf [47,48]. Conversely, the atrial myocardium becomes pectinate in structure by about 14 dpf [46,49]. Genetic lineage tracing has shown that pectinate atrial cardiomyocytes in the mature atrium form through the proliferation and branching of existing cardiomyocytes [49], suggesting that there are different mechanisms driving the maturation of the atrial and ventricular chambers. Thus, the heart undergoes extensive morphological changes throughout development that establish each of the cardiac chambers.

### 2.2. Signals Regulating the Specification of Atrial Progenitors

**Figure 2:**
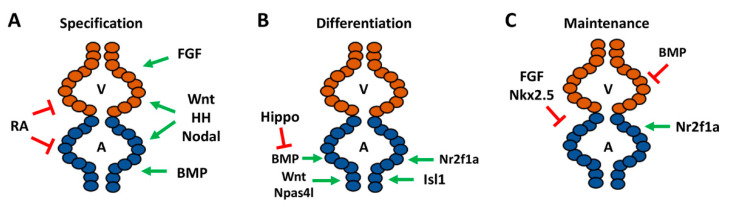
Signaling pathways required for different stages of atrial development and cardiac maintenance in zebrafish. (**A**) Pathways required for specification of chamber progenitors in the early embryo. (**B**) Factors responsible for differentiation of atrial cardiomyocytes within the ALPM and at the venous pole. (**C**) Genes shown to promote or repress atrial identity within differentiated embryonic ventricular cardiomyocytes and repress ventricular gene expression in atrial cardiomyocytes. A—atrium, V—ventricle.

Numerous early signaling pathways are involved in the regulation of cardiomyocyte specification as part of their requirements in the establishment of the body plan, which factors into determination of the number of atrial cardiomyocytes within the zebrafish heart. Signals including Hedgehog (HH), canonical Wnt/β-catenin (Wnt), and Nodal exert similar effects promoting the specification of both atrial and ventricular cardiomyocytes. Increasing HH signaling through the injection of *sonic hedgehog* mRNA promotes atrial and ventricular specification and yields hearts with increased cardiomyocytes, while loss of HH signaling through the genetic and pharmacological inhibition of the Smoothened receptor reduces cardiac progenitor specification, consequently resulting in hearts with decreased atrial and ventricular cardiomyocytes [50]. Similarly, using heat-shock inducible transgenes to increase and decrease canonical Wnt signaling by the initiation of gastrulation leads to corresponding increases and decreases in cardiomyocyte progenitor specification, respectively [51,52], due to effects on the induction of mesoderm. Nodal signaling, which is required for mesoderm specification, is also required for cardiomyocyte specification. Specifically, mutations in the Apelin receptor, which modulates Nodal signaling, also lead to a loss of both atrial and ventricular cardiomyocytes [53,54] (Figure 2A).

Although initial studies in mice and zebrafish concluded that retinoic acid (RA) signaling promotes atrial cardiomyocyte specification [55,56], pivotal work in zebrafish showed that its earliest requirement is to restrict cardiac specification of both atrial and ventricular progenitors within the ALPM [57,58]. Specifically, inhibition of RA signaling through use of the *neckless* (*nls*) mutant, which harbors a mutation in the major embryonic producer of RA *aldh1a2* (formerly called *raldh2*), and treatment with RA signaling antagonists results in enlarged hearts with an increase in the number of atrial and ventricular cardiomyocytes [57,58]. Excess RA signaling can also inhibit the specification of both atrial and ventricular cardiomyocytes, while intermediate increases in RA signaling can differentially affect the specification of cardiomyocytes, which likely led to the initial interpretations that RA signaling promotes atrial specification [59,60]. Thus, the specification of both atrial and ventricular progenitors is effectively balanced through opposing roles of early HH, Wnt, and Nodal, which promote cardiomyocyte specification, and the restrictive role of RA signaling (Figure 2A).

In contrast to signals that have similar consequences on the specification of atrial and ventricular cardiomyocytes, early signals that affect dorsal–ventral patterning have differential effects on chamber specification. The dorsalizing pathway fibroblast growth factor (FGF) signaling preferentially affects the specification of ventricular progenitors with minimal effect on the atrium [61]. Conversely, the ventralizing bone morphogenic protein (BMP) signaling pathway is necessary and sufficient to promote atrial specification. Loss of BMP signaling in the *alk8/lost-a-fin* (*laf*) mutants, treatment with pharmacological BMP signaling inhibitors, and an inducible dominant-negative Alk8 transgene all demonstrated that BMP signaling is required early for atrial progenitor specification during gastrulation and results in hearts with decreased atrial cardiomyocytes. Consistently, increasing BMP signaling through the injection of constitutively-active *alk8* mRNA results in increased atrial cardiomyocytes, reinforcing the role of BMP in atrial specification [62,63]. Thus, early signaling that ventralizes the embryo is required for the specification of atrial precursors (Figure 2A).

### 2.3. Signals Directing the Differentiation of Atrial Cardiomyocytes

Subsequent to the signals that modulate atrial cardiomyocyte number and chamber size through establishment of cardiomyocyte progenitor populations, signals then control atrial chamber size through influencing cardiomyocyte differentiation from within the ALPM. While RA signaling may restrict the number of both atrial and ventricular cardiomyocytes, the lineage tracing analysis suggested that signals downstream of RA may differentially affect atrial and ventricular productions [58]. Depletion of *hoxb5b*, an RA-responsive gene within the ALPM which is posterior to the cardiac progenitors, has been shown to result in excess atrial cardiomyocytes, suggesting it may function downstream of RA to restrict the number of atrial cardiomyocytes [58]. However, these results require confirmation with genetic mutants. Additionally, *cloche* mutants, which we now understand are caused by mutation of the transcription factor *Npas4l* [64], and downstream factors *Scl* (Tal1) and *Etv2* (Etsrp) function to restrict heart size through promoting hematopoietic and vascular lineages within the ALPM. *Cloche* mutants or concurrent depletion of *Scl* and *Etv2* with morpholinos both produce enlarged hearts due to an increase in atrial cardiomyocytes [65] (Figure 2B). In addition to its early role, canonical Wnt signaling functions reiteratively in later roles. At the tailbud stage, excess Wnt signaling can potently inhibit the differentiation, but not the specification, of both atrial and ventricular progenitors [51]. However, in contrast to earlier requirements that have similar effects on cardiomyocyte progenitors, during later stages of somitogenesis Wnt signaling specifically affects the differentiation of atrial cardiomyocytes. Using heat-shock inducible transgenes to modulate Wnt signaling at the 16 somite stage, when the cardiac progenitors reside within bilateral populations in the ALPM, showed that increased Wnt signaling produces an increase in the number of atrial cardiomyocytes and decreased Wnt signaling produces fewer atrial cardiomyocytes [51]. Similarly, while BMP signaling is predominantly required for atrial cardiomyocyte specification throughout gastrulation, it has also been shown to play reiterative roles in atrial differentiation. Blocking BMP signaling using a heat-shock inducible *Noggin3* transgene at 16 hpf causes a specific decrease in atrial cardiomyocytes that is independent of its earlier role ventralizing embryos [63]. In contrast to the positive influences of Wnt and BMP signaling on atrial differentiation during late somitogenesis, research has shown that, at these stages, Hippo signaling restricts the number of atrial cardiomyocytes added to the venous pole. Large tumor suppressor kinase (Lats) proteins are critical regulators of the Hippo pathway [66], because activation of Lats1/2 is required for proper Hippo signaling. In *lats1*; *lats2* mutants, it was shown that decreased Hippo signaling results in an increased number of atrial cardiomyocytes, supporting that Hippo signaling restricts atrial size. Subsequent analysis revealed that Hippo functions upstream of BMP signaling to restrict the number of SHF-derived cardiomyocytes that arise from the ALPM and contribute to the venous pole of the heart [67] (Figure 2B), providing insight into the interplay between these signaling pathways in heart development.

In addition to the aforementioned signaling pathways, specific members of the Islet (*Isl*) and Nuclear receptor 2 (*Nr2f*/Coup-tf) transcription factor families have been shown to be necessary to promote the differentiation of atrial cardiomyocytes at the venous pole. Isl1 marks the venous SHF in zebrafish. Consistent with this expression, *isl1* mutants have decreased atrial cardiomyocytes, which temporal differentiation assays have shown to be due to reduced differentiation [41] (Figure 2B). Interestingly, *Isl1* is specifically required for the differentiation of cardiomyocytes at the venous pole in zebrafish embryos, contrasting with the requirement of *Isl1* in mice, which is required for the differentiation of all SHF progenitors at both the posterior venous and anterior arterial poles [41,44]. However, it has been shown that *isl2b* is required for development of the arterial pole in zebrafish, suggesting that in teleosts Islet family members have distinct roles in progenitor differentiation at the different poles of the heart [68].

Nr2f transcription factors are the proteins that are most prominently associated with the determination of atrial identity in vertebrates. Global knockout of *Nr2f2* in mice results in embryos with small, dysmorphic atria [69]. In zebrafish, our lab has shown that Nr2f1a is the functional equivalent of mammalian *Nr2f2* with respect to heart development. Within the embryonic zebrafish heart, *Nr2f1a* is expressed specifically within atrial cardiomyocytes and progenitors at the venous pole. *Nr2f1a* mutant zebrafish develop smaller atria due to decreased differentiation of atrial cardiomyocytes at the venous pole [70]. Thus, Wnt, BMP, and Hippo signaling and the *Hoxb5b*, *Isl1*, and *Nr2f1a* transcription factors all influence the proper differentiation of atrial cardiomyocytes within the zebrafish heart (Figure 2B).

### 2.4. Maintenance and Plasticity of Cardiomyocyte Identity

Although the lineage tracing of blastula cell embryos has suggested that normally the fates of cardiomyocytes are largely distinct [35], studies have indicated that there is plasticity between atrial and ventricular progenitors and that chamber identity must be continually reinforced in zebrafish embryos after the cardiomyocytes have overtly differentiated [25,27,28,71,72,73,74]. A clue that there is greater plasticity between atrial and ventricular cardiomyocytes in normal zebrafish heart development was indicated from genetic labeling of embryonic cardiomyocytes with *amhc:Cre^ERT2^* and loxP-mediated color-switch reporter transgenes [49]. Some *amhc*-labeled cardiomyocytes within the embryos differentiate as ventricular cardiomyocytes, suggesting that there are initially cardiomyocytes which have begun to differentiate with the potential to become either atrial or ventricular cardiomyocytes, but then differentiate as ventricular cardiomyocytes as the embryonic heart matures [49]. The necessity to maintain ventricular identity at the expense of atrial identity is illustrated by the *Nkx2.5* and *Nkx2.7* transcription factors [73,75]. Zebrafish *nkx2.5* mutants initially have an equivalent number of atrial and ventricular cardiomyocytes as their wild-type siblings. However, they progressively develop an enlarged atrium and diminutive ventricle as the ventricular cardiomyocytes take on atrial identity (Figure 2C). While *Nkx2.5* is the primary factor necessary to maintain ventricular identity, concomitant loss of *Nkx2.7* has been shown to exacerbate the fate transformation, suggesting some limited redundancy between these transcription factors. *Nkx2.5/7* maintain ventricular identity by promoting the expression of ventricular-specific genes, *irx4* and *hey2* [73,75]. Remarkably, induction of *Nkx2.5* with a heat-shock inducible transgene at late somitogenesis is sufficient to rescue *Nkx2.5* mutants to adulthood, which suggests that the requirement of *Nkx2.5* in repressing atrial identity in ventricular cardiomyocytes occurs within a specific developmental window, but that it does not perform this role throughout life [75].

*Fgf8a* is expressed within the zebrafish ventricle [76]. It has been shown that the FGF pathway sits at the top of a signaling cascade to reinforce ventricular identity at the expense of atrial identity [74]. Inhibition of FGF signaling with a dominant negative transgene or pharmacological inhibitors beginning at the 18 somite stage results in ectopic atrial gene expression within the ventricle [74]. Interestingly, it was shown that overexpression of *Nkx2.5* in embryos while concurrently inhibiting FGF signaling resulted in a partial rescue of this fate transformation, indicating that FGF signaling functions upstream of *Nkx2.5* in maintaining ventricular identity (Figure 2C), but that other unidentified factors likely also play a role in the repression of atrial identity within ventricular cardiomyocytes [74]. While FGF signaling is necessary to maintain ventricular identity, BMP signaling may also need to be actively repressed in ventricular cardiomyocytes to prevent the acquisition of atrial cardiomyocyte identity (Figure 2C). Increasing BMP signaling at the 18 somite stage with a heat-shock inducible BMP2b in embryos depleted of *smad6a*, which represses BMP signaling, results in both fewer ventricular cardiomyocytes and some ventricular cardiomyocytes that ectopically express Amhc [63]. However, the mechanisms by which excess BMP signaling promotes atrial identity in the ventricle still needs to be elucidated. The plasticity between atrial and ventricular cardiomyocyte identities can also be seen in the regenerative response to cardiac injury in embryonic zebrafish hearts. Upon ablation of ventricular cardiomyocytes in 5 dpf larval zebrafish, atrial cardiomyocytes proliferate and transdifferentiate into ventricular cardiomyocytes to regenerate the ventricle [77]. This atrial-to-ventricular injury response is diminished in adult zebrafish, suggesting that, like the requirement of *Nkx2.5* in maintaining ventricular identity, there is an age-dependent developmental window to this plasticity [77].

Despite the evidence that factors maintain ventricular identity in embryonic zebrafish hearts, it is not known if there are genes responsible for maintaining atrial identity. Thus far, factors have not been identified that clearly show differentiated atrial cardiomyocytes need to repress ventricular cardiomyocyte identity in zebrafish. However, *Nr2f* transcription factors have been shown to maintain atrial cardiomyocyte identity in mice, in addition to their requirement in atrial cardiomyocyte differentiation. In mice, cardiac-specific knockout of *Nr2f2* with Myh6-Cre results in ectopic expression of ventricular genes within atrial cardiomyocytes, because *Nr2f2* represses ventricular-identity genes *Irx4* and *Hey2* [78]. Consistently, overexpression of *Nr2f2* was sufficient to confer atrial identity within ventricular cardiomyocytes [78]. In addition to reduced atrial cardiomyocyte number, *nr2f1a* mutant zebrafish display an expansion of AVC markers into the atrium, suggesting that *Nr2f1a* is required to limit the size of the AVC [70]. *Nr2f1a* mutants also have an expansion of the ventricular differentiation marker *vmhc* into the atrium (Figure 2C), although there was no apparent effect on ventricular cardiomyocyte number at least through 48 hpf [70]. It is not clear, however, that these effects represent a transdifferentiation of the atrial cardiomyocytes comparable to what is found in mice. Additional studies are needed to determine if atrial cardiomyocytes in *Nr2f1a* mutant zebrafish undergo the same fate transformation that is seen in the *Nr2f2* conditional knockout mice at later stages of development. Overall, these studies have shown that differentiated embryonic cardiomyocytes in zebrafish maintain a degree of plasticity and the ventricle will adopt atrial identity in the absence of critical maintenance factors (Figure 2C).

### 2.5. Conserved Transcriptional Networks Promoting Sinoatrial Node Development

The zebrafish heart first begins beating at 24 hpf with the formation of the linear heart tube. Initial contractions are slow and peristaltic, originating at the venous pole. As the heart loops, the electrophysiology changes and a conduction delay in the AVC leads to the sequential atrial–ventricular contraction pattern [22]. With the use of optogenetics, it has been shown that the initial pacemaker region is a large area at the venous pole of the heart tube, which then condenses to form a ring by 48 hpf (Figure 1E,F). By 72 hpf, the dominant pacemaker has narrowed further to a small population of cells in the inner curvature of the atrium [79] (Figure 1G). Interestingly, this location is analogous to the location of the pacemaker in the right atrium of the mammalian heart.

The genetic mechanisms underlying development of the SAN have been extensively studied in mice, but thus far they have not been as well characterized in zebrafish. We will cover specifically what is known about the development of the SAN in zebrafish in comparison to mammals. Multiple reviews have already detailed the development of the cardiac conduction system as a whole [31,80,81]. Work in mice has shown that information from a complex genetic network of transcription factors is integrated to establish SAN identity within the atrial myocardium at the venous pole. T-box transcription factors *Tbx3* and *Tbx18* have been shown to be vital for proper SAN development, although they have different functions [82]. *Tbx18* deficient mice develop a severely hypoplastic SAN, however the SAN gene program is maintained within these cells and the mice do not develop overt bradycardia. Conversely, *Tbx3* deficiency leads to a morphologically normal SAN that ectopically expresses atrial myocardium genes. This suggests that *Tbx18* is required for SAN morphogenesis and *Tbx3* is required for pacemaker differentiation [82]. Interestingly, overexpression of *Tbx3* in the mouse atrial myocardium is sufficient to confer pacemaker identity, highlighting its role as a key driver of SAN identity [83]. In contrast to *Tbx3* and *Tbx18*, *Nkx2.5* represses SAN identity within atrial cardiomyocytes. *Nkx2.5* mutant mice have an enlarged SAN, while *Nkx2.5* overexpression leads to SAN hypoplasia and decreased expression of *Tbx3* [84,85,86]. *Isl1* and *Shox2* have also been shown to be expressed in and required for pacemaker development [87,88,89,90,91]. Loss of function of either of these transcription factors results in a hypoplastic SAN and decreased expression of *Tbx3* [90,91]. *Shox2* also functions upstream of *Nkx2.5*, with *Shox2* mutants developing ectopic expression of *Nkx2.5* within the pacemaker. Overexpression of Shox2 is also able to partially repress *Nkx2.5* [84,89,91,92]. Thus, these studies in mice have established a multi-tier genetic network in which cross regulation of the transcription factors *Tbx3*, *Isl1*, and *Shox2* promote SAN identity and oppose the repressive action of *Nkx2.5* to confer SAN identity within a proper number of atrial cardiomyocytes at the venous pole of the heart.

### 2.6. Development of the Zebrafish Sinoatrial Node

**Figure 3:**
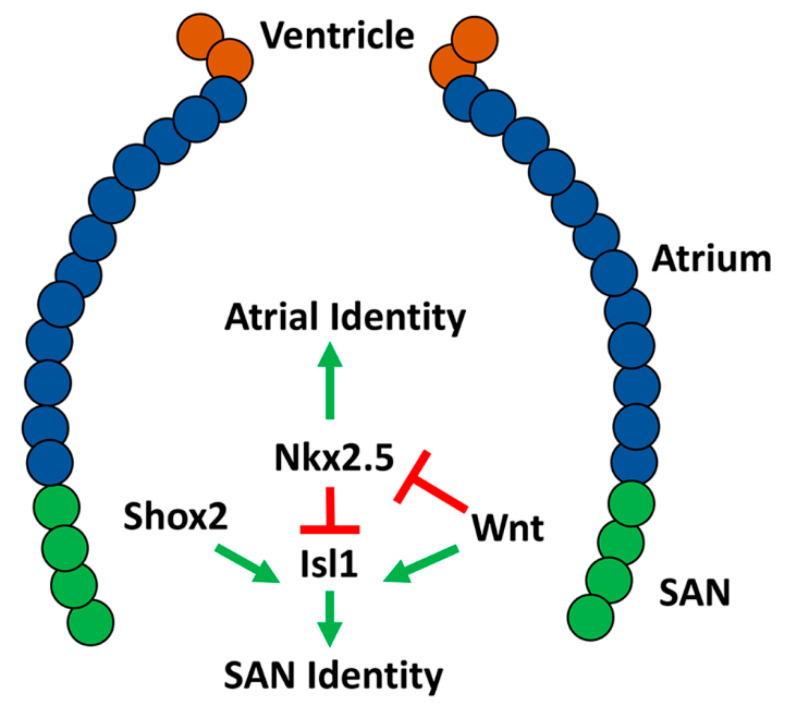
Molecular network shown to be responsible for differentiation of the sinoatrial node (SAN) in embryonic zebrafish.

While earlier studies in zebrafish indicated the presence of specialized pacemaker tissue in the heart [22,79], the molecular regulation of the zebrafish SAN has historically been largely understudied, in part due to the lack of reliable SAN markers. Isl1 was the first molecular marker identified for the zebrafish SAN. Isl1-expressing cells form a ring at the venous pole of the atrium in embryonic hearts [26]. Its expression is maintained in a ring at the base of the atrium and junction with the sinus venosus through adulthood. In addition to reduced atrial cardiomyocytes, *isl1* mutants have an abnormal heartbeat, highlighting its conserved importance in pacemaker development and function [26,41]. It has been indicated that Isl1 promotes SAN development upstream of Wnt/β-catenin, because Wnt signaling is lost in the SAN of *isl1* mutants [93]. Furthermore, at these later stages, Wnt signaling controls heart rate through modulating the response of the pacemaker cardiomyocytes to parasympathetic input [93]. In contrast to mice, which have lost Shox and only have *Shox2*, humans and zebrafish have retained both *Shox* and *Shox2*. Interestingly, by replacing *Shox2* with Shox in mice, it has been shown that human Shox and mouse *Shox2* can similarly promote SAN development [92]. In zebrafish, *Shox2* has been shown to promote SAN identity, with *shox2* morpholino-depleted zebrafish developing bradycardia [89]. Overexpression of Isl1 in *Shox2* morphants can rescue the bradycardia phenotype, suggesting that *Shox2* functions upstream of Isl1 in SAN development [94] (Figure 3). However, the requirement of *Shox2* in zebrafish and the relationship to Isl1 within the SAN should be verified through the use of genetic mutants. Consistent with conserved requirements for *Nkx2.5* repressing SAN identity within working atrial cardiomyocytes, studies have shown that Nkx2.5 limits Isl1 expression to the venous pole of the atrium [95] (Figure 3). *Nkx2.5* zebrafish mutants have a progressive expansion of *Isl1+* atrial cardiomyocytes. Conversely, *Nkx2.5* overexpression leads to diminished *Isl1* expression at the venous pole [84,85,95]. *Nkx2.5* expression is largely absent from the SAN cells, although *Nkx2.5* is expressed throughout the zebrafish heart at earlier stages; therefore, it was questioned if the *Nkx2.5*–SAN cardiomyocytes are derived from an *Nkx2.5+* progenitor population [96,97]. Lineage tracing in zebrafish embryos confirmed that the pacemaker cardiomyocytes are derived from *Nkx2.5+* progenitors that come from the most lateral regions of the cardiac mesoderm [98]. Furthermore, in this population, *Wnt5b* initiates canonical Wnt signaling, which silences *Nkx2.5* and activates *Isl1* and *Tbx18* (Figure 3). Consistent with this role in cardiomyocyte specification, loss of *Wnt5b* function results in a decrease in pacemaker cardiomyocytes and corresponding increase in the number of atrial cardiomyocytes. Conversely, overexpression of *Wnt5b* produces the opposite effect, with increased pacemaker cardiomyocytes and decreased atrial cardiomyocytes [98]. Interestingly, *Wnt5b* mutant embryos develop slower heart rates compared to wild-type, however inhibition of Wnt signaling by overexpressing *Axin1* between 36 and 52 hpf results in an increase in heart rate, emphasizing that canonical Wnt signaling first helps establish the SAN earlier in cardiogenesis and then modulates pacemaker function later [93,98]. Consistently, while previous work did not investigate the differentiation of pacemaker cells specifically, the early requirement of *Wtn5b* is consistent with the previously identified role for Wnt signaling in promoting the differentiation of venous atrial cardiomyocytes [51]. Canonical Wnt signaling as a driver of SAN identity has also been shown in mouse and human embryonic stem cells, indicating its conserved role in pacemaker cardiomyocyte differentiation [99]. Thus, while investigation of the genetic networks driving SAN formation in zebrafish to this point is less extensive than mice, the current data support that a fundamentally conserved network of transcription factors are necessary to establish the proper number of pacemaker cardiomyocytes within the venous pole of the zebrafish heart (Figure 3).

### 2.7. Zebrafish as a Model for Human Atrial and SAN Defects

Despite the different number of atrial and ventricular chambers within their heart, the high degree of genetic conservation regulating early cardiac patterning and cardiomyocyte function allows studies in zebrafish to provide insights into the molecular etiology of human CHDs. While mutations that affect patterning of the atrium in zebrafish embryos can perturb the size of the atrium, improper specification of the atrium in humans is often associated with atrial septal defects (ASDs) and atrioventricular septal defects (AVSDs). AVSDs, a subtype of ASD, account for 5–7% of CHDs, while ASDs are reported in as many as 20% of CHD patients [5,6,7,8,9]. ASDs are also the most likely CHD to be newly diagnosed in adults, comprising 20–40% of adult CHD diagnoses [100]. Furthermore, septal defects, including ASDs, AVSDs, and ventricular septal defects (VSDs), account for the majority (35–54%) of adult CHD-related hospitalizations [101,102]. With regard to SAN development, improper specification of the SAN in humans can manifest as sick sinus syndrome, arrhythmias, and atrial fibrillation [11,12,13,14,103,104,105,106]. Importantly, ASDs are often associated with arrhythmias and conduction defects, which can arise as a consequence of the structural defect itself or occur concurrently as a result of genetic mutations in genes responsible for development of both the atrium and the SAN [10,107,108]. This is an important distinction because surgical intervention can often correct the structural malformations, but an underlying conduction defect may remain and cause life threatening heart problems later in life, with arrhythmias being a leading cause of mortality in adults with CHDs [10,108,109].

Mutations in *TBX5*, HH signaling, *NR2F2*, and *NKX2.5* are some of the specific and better studied genes associated with ASDs and AVSDs in humans. Murine models have highlighted that improper specification of the venous SHF, leading to loss of the dorsal mesenchyme protrusion, is a primary cause of ASDs [110]. In humans, mutations in TBX5, which underlie Holt–Oram syndrome, and the Smoothened receptor, which is required for HH signaling, both result in a spectrum of similar developmental defects that include ASDs and AVSDs [10,111]. Is has been shown that Tbx5 functions upstream of HH signaling in the posterior SHF. Haploinsufficient *Tbx5* mutant mice develop AVSDs, which can be rescued with restoration of HH signaling [112,113]. *Tbx5a* mutant zebrafish, called *heartstrings*, have distended hearts, although cardiac specification does not appear to be affected [114] (Table 1). As illustrated above, one of the consequences of loss of HH signaling is decreased atrial chamber size in zebrafish [50]. Interestingly, RA signaling also regulates *Tbx5* expression within the posterior SHF [115]. As mentioned above, RA signaling restricts cardiomyocyte progenitor specification [57,58]. RA-deficient mice have a posterior expansion of the SHF, similar to the posterior expansion found in zebrafish embryos [116,117]. Importantly, RA signaling is required to promote *Tbx5* expression and repress *Tbx1* expression. Blocking RA signaling at the time point when *Tbx5* is required in the SHF results in AVSDs [115].

*NR2F*1 and *NR2F2* are both expressed in human atrial cardiomyocytes [128]. However, to this point, only mutations in *NR2F2* in humans have been associated with CHDs [1,2,119,120,129,130] (Table 1). NR2F1 and NF2F2 are required for the differentiation of atrial cardiomyocytes in human embryonic stem cells (hESCs) [128]. Furthermore, work in hESCs has also shown that *NR2F1* is antagonized by *ISL1* downstream of RA signaling in the differentiation of atrial cardiomyocytes [131]. In mice, while *Nr2f2* is required for atrial development and maintenance, the mechanisms underlying the ASDs and AVSDs in humans have not been elucidated [69,78]. *Nr2f1a* single mutant zebrafish have improper atrial cardiomyocyte differentiation at the venous pole, implying that one mechanism underlying ASDs in mammals is a failure of posterior SHF cells to differentiate. However, *nr2f1a* mutants also have an expanded AVC [70], which we posit could lead to AVSDs from improper alignment and valve specification. Interestingly, although *Nr2fs* have historically been associated with atrial development, human *NR2F2* mutations are also associated with CHDs affecting the ventricle, including VSDs, left ventricular outflow tract obstruction (LVOTO), and double outlet right ventricle (DORV) [1,2,119,120]. Additionally, mutations in *NR2F1* and *NR2F2* have been associated with craniofacial defects in humans [120,130]. Interestingly, our work has revealed that zebrafish *Nr2f1a* and *Nr2f2* function redundantly to restrict the number of ventricular cardiomyocytes and promote pharyngeal muscle specification at earlier stages, but do not overtly function redundantly in promoting atrial cardiomyocyte differentiation [118]. Thus, understanding the dose dependency of *nr2f* genes in zebrafish may provide an understanding of the variability of CHDs affecting both the atrium and ventricle, and craniofacial defects associated with NR2F mutations in humans.

*Nkx2.5* haploinsufficiency in humans is associated with ASDs, AVSDs, and tetralogy of Fallot (ToF), as well as conduction defects, such as atrial fibrillation, arrhythmias, and atrioventricular (AV) block [3,4,15,108,121,123,132,133], consistent with the multiple roles *Nkx2.5* plays during cardiogenesis (Table 1). As illustrated above, *Nkx2.5* has conserved requirements in the development of both the SAN and the working myocardium in vertebrates. *Nkx2.5* knockout mice are embryonic lethal with abnormal heart development [134,135]. Importantly, atrial-specific knockout of *Nkx2.5* in mice results in ASDs and an enlargement of the SAN [85], reminiscent of the atrial and SAN expansions due to *nkx2.5* loss in zebrafish hearts [95]. Interestingly, *Nkx2.5* knock-in mouse models have been shown to reproduce the phenotype reported in patients harboring the same mutation. Heterozygous knock-in mice have been reported to develop ASDs, AV block, and arrhythmias, the last of which is thought to be due to ectopic pacemaker activity within the atrium [103,136,137]. Consistent with the conservation of core regulators of SAN development, *SHOX2* mutations have been reported in patients with atrial fibrillation [13,105,106] (Table 1). Global *Shox2* knockout mice are embryonic lethal and develop a hypoplastic SAN [89]. Additionally, as mentioned previously, *Shox2* functions upstream of *Nkx2.5* during pacemaker specification and promotes SAN development, with Shox2-depleted zebrafish developing bradycardia [89,94].

An additional SAN marker that has been modeled in zebrafish is the ion channel *HCN4*, which lies downstream of the SAN regulatory genes mentioned above and is vital for normal pacemaker function [30]. Mutations in *HCN4* are associated with sick sinus syndrome in humans [14,104] (Table 1). Global and cardiac-specific *Hcn4* knockout mice are embryonic lethal due to a lack of functional pacemaker cells [138], and knockdown of *Hcn4* in adult mice leads to bradycardia and eventual heart block [139]. In zebrafish, it has been shown that morpholino-based knockdown of *hcn4* causes bradycardia and sinus pauses reminiscent of the sick sinus syndrome phenotypes found in patients with *HCN4* mutations [127]. Therefore, zebrafish can further our understanding of CHDs that are caused by mutations in vital regulatory genes, such as *Nkx2.5* and *Shox2*, as well as their downstream targets, such as *Hcn4*.

## 3. Conclusions

Zebrafish are an excellent model for dissecting the molecular and genetic mechanisms of vertebrate heart development. Contemporary advances through the use of zebrafish have furthered our understanding of conserved factors driving the development and maintenance of the vertebrate atrium and SAN. Mutations in genes that are required for the specification, differentiation, and maintenance of venous pole cardiomyocytes are often associated with a spectrum of CHDs affecting the atria in humans. While numerous factors have been shown to be involved in the development of the vertebrate atrium and SAN, how these signaling pathways interact and are coordinated to promote proper cardiogenesis and maintain cardiomyocyte identity require further investigation. Future work in zebrafish will help to dissect the critical mechanisms and regulatory networks governing venous pole development in the vertebrate heart, providing insights in the etiology of CHDs in humans.

## Figures and Tables

**Table 1:** Genes associated with atrial and SAN defects in humans and their zebrafish orthologs. ASD—atrial septal defect; AVSD—atrioventricular septal defect; DORV—double outlet right ventricle; LVOTO—left ventricular outflow tract obstruction; ToF—tetralogy of Fallot; VSD—ventricular septal defect.

Human Gene	Human CHD	Zebrafish Gene(s)	Zebrafish Phenotype	References
*NR2F2*	AVSD, ASD, LVOTO, DORV, VSD	*nr2f1a*, *nr2f2*	*Nr2f1a*: Atrial differentiation defects *Nr2f1a*; *Nr2f2*: Ventricular specification defects	[1,2,70,118,119,120]
*NKX2.5*	ASD, VSD, ToF, Conduction defects	*nkx2.5*	Defects in cardiomyocyte proliferation, differentiation, and maintenance, Conduction defects	[3,4,73,108,121,122,123,124]
*TBX5*	ASD, VSD, Holt–Oram Syndrome	*tbx5a*, *tbx5b*	Looping defects, Bradycardia	[10,114,125,126]
*SHOX2*	Atrial Fibrillation	*shox2*	Bradycardia	[13,94,105,106]
*HCN4*	Sick Sinus Syndrome	*hcn4*	Bradycardia	[14,104,127]

## Data Availability

Not applicable.
